# RBM15-mediated metabolic reprogramming boosts immune response in colorectal cancer

**DOI:** 10.3389/fimmu.2025.1515568

**Published:** 2025-04-30

**Authors:** Chen Wang, Mengyan Chen, Panyu Chen, Jinlu Han, Hong Hu, Jiong Chen, Qiong Wu, De Zhao, Tongshuai Wang, Jingyi Zhou, Qi Li, Runkai Zhou, Yugang Wen, Jing Yang, Min Shi, Yugang Wang

**Affiliations:** ^1^ Department of Gastroenterology, Tongren Hospital, Shanghai Jiao Tong University School of Medicine, Shanghai, China; ^2^ Hongqiao International Institute of Medicine, Tongren Hospital, Shanghai Jiao Tong University School of Medicine, Shanghai, China; ^3^ Key Laboratory for Translational Research and Innovative Therapeutics of Gastrointestinal Oncology, Tongren Hospital, Shanghai Jiao Tong University School of Medicine, Shanghai, China; ^4^ State Key Laboratory of Drug Research, Shanghai Institute of Materia Medica, Chinese Academy of Sciences, Shanghai, China; ^5^ Department of Oncology, Shanghai General Hospital, Shanghai Jiao Tong University School of Medicine, Shanghai, China; ^6^ Department of General Surgery, Shanghai General Hospital, Shanghai Jiao Tong University School of Medicine, Shanghai, China; ^7^ Department of Pathology, Tongren Hospital, Shanghai Jiao Tong University School of Medicine, Shanghai, China

**Keywords:** colorectal cancer, RBM15, microenvironment, RNA modification, m^6^A

## Abstract

**Introduction:**

Immune checkpoint blockade (ICB) therapy has shown promise in treating advanced colorectal cancer, particularly in patients with microsatellite instability-high (MSI-H) tumors. However, only a subset of these patients responds favorably, highlighting the need for strategies to improve immunotherapy efficacy.

**Methods:**

To identify potential regulators of immunotherapy response, we conducted a comprehensive analysis of colorectal cancer datasets from The Cancer Genome Atlas (TCGA). We performed multi-omics analyses and functional assays in both human and murine colorectal cancer cell lines. Additionally, we evaluated tumor growth and immune cell infiltration using syngeneic mouse models.

**Results:**

Our analysis revealed that RNA binding motif protein 15 (RBM15) is highly expressed in colorectal cancer and correlates with poor patient prognosis. Functional studies demonstrated that RBM15 loss led to increased expression of fumarate hydratase (FH). This led to decreased levels of fumarate, a metabolite known to suppress anti-tumor immune responses. *In vivo*, RBM15 depletion significantly delayed tumor progression and enhanced CD8⁺ T cell infiltration and activation in the tumor microenvironment.

**Discussion:**

These findings identify RBM15 as a negative regulator of anti-tumor immunity in colorectal cancer. Targeting RBM15 may represent a novel strategy to boost immune responsiveness and improve outcomes for patients undergoing immunotherapy.

## Introduction

Colorectal cancer (CRC) poses a significant clinical challenge worldwide, with a 5-year survival rate of less than 13% for patients with advanced diseases (1, 2). While immunotherapies open up therapeutic opportunities to advanced CRC, their effectiveness remains limited (3). Only 10-15% patients with microsatellite instability-high (MSI-H) tumors respond to immune checkpoint inhibitors (ICIs) such as anti-PD-1 antibodies (4). Moreover, clinical responses to immunotherapies are generally incomplete and not durable, due in part to high tumor heterogeneity and an immunosuppressive tumor microenvironment (3, 5). Therefore, there is an urgent need to identify novel therapeutic strategies to sensitize immunotherapy.

In recent years, the role of RBM15 in various cancers has attracted significant attention. Studies have shown that inhibition of RBM15 can promote macrophage infiltration and enhance its phagocytic activity toward pancreatic cancer cells. Moreover, RBM15 collaborates with methyltransferase 3 (METTL3) to upregulate N6-methyladenosine (m^6^A) modification of long non-coding RNAs, facilitating bladder cancer initiation and progression2. Additionally, RBM15 regulates m^6^A methylation to upregulate integrin subunit beta like 1 (ITGBL1) expression, promoting the progression of colorectal adenocarcinoma. RBM15 also modulates procollagen-lysine,2-oxoglutarate 5-dioxygenase 3 (PLOD3), enhancing tumor-infiltrating CD4+ T cell abundance in esophageal squamous cell carcinoma (ESCC), correlating with favorable prognosis in ESCC4. Furthermore, RBM15 may promote malignant progression and immune escape in breast cancer cells by regulating the stability of karyopherin subunit alpha 2 (KPNA2) mRNA5.

N^6^-Methyladenosine (m^6^A) is the most abundant and conserved modification of eukaryotic mRNAs, and its role in tumor immunomodulation has become a focus of extensive research (6, 7). For example, the m^6^A reader protein YTHDF1 drives immune evasion and resistance to immunotherapies by promoting the degradation of major histocompatibility complex class I (MHC-I) (8). RNA binding motif protein 15 (RBM15) is a crucial regulator of m^6^A modification (9). RBM15 interacts with the m^6^A writer complex and positively regulates m^6^A levels, influencing on alternative splicing and mRNA stability (10). While ample evidence supports the role of RBM15 in oncogenesis, these studies primarily focus on alterations in cancer cells within immunocompromised environments (11–13). Little is known about whether or how RBM15 regulates tumor immune surveillance in cancers.

Accumulating studies indicates that metabolic rewiring in malignant cells impairs both innate and adaptive immune functions, thus promoting tumor progression (14). Cancer cell-intrinsic and cancer cell-extrinsic mechanisms both play crucial roles in tumor immune evasion and responses. For example, cancer cells compete with CD8+ cytotoxic T lymphocytes (CTLs) for glucose uptake to meet their increased proliferative demands, which compromises CTL function (15). In addition, the degradation of extracellular ATP by the ectonucleotidases CD39 and CD73 generates adenosine, which induces an immunosuppressive tumor microenvironment by reducing dendritic cell (DC) recruitment (16). However, whether RBM15 plays a role in metabolic reprogramming that could affect anti-tumor immunomodulation remains poorly understood.

In this study, we reveal a cancer cell-intrinsic function of RBM15 in driving immune evasion in colorectal cancer. We found that RBM15 is overexpressed in colorectal cancer and is associated with poor prognosis. RBM15 deficiency restrains tumor growth by enhancing immune cell infiltration. Mechanistically, RBM15 depletion increases the expression of fumarate hydratase (FH), which in turn decreases the level of fumarate, a known suppressor of anti-tumor immunity. Overall, our study identifies RBM15 as a potent suppressor of anti-tumor immunity and highlights RBM15 as a promising therapeutic target for restoring immune surveillance in colorectal cancer.

## Results

### RBM15 overexpression correlates with reduced immune cell infiltration in colorectal cancer

**Figure 1:**
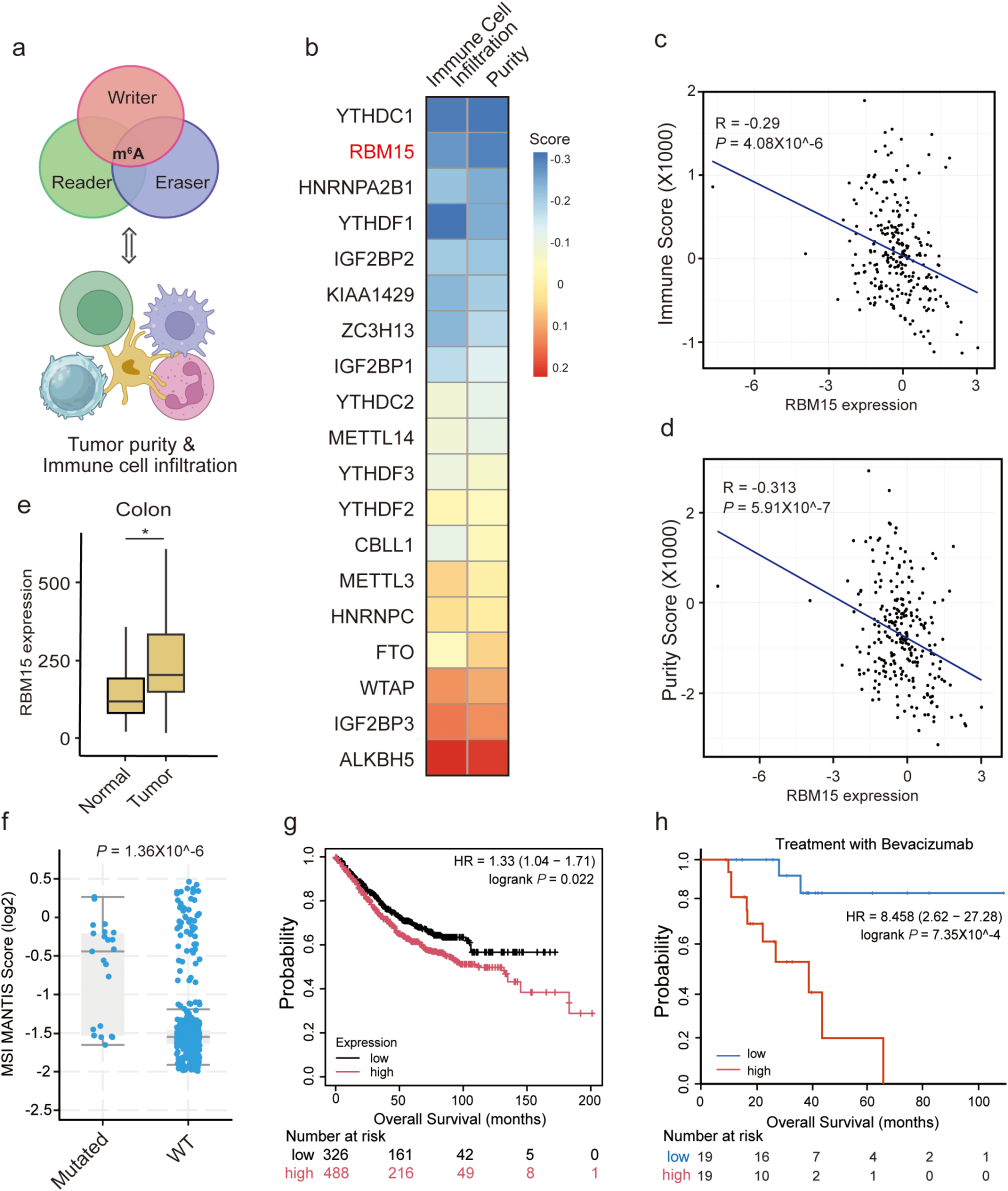
RBM15 overexpression is negatively correlated with immune cell infiltration in colorectal cancer. **(a)** A conceptual diagram illustrating the strategy to identify potential correlation between the expression levels of m^6^A regulators and tumor purity, as well as immune cell infiltration. **(b)** Heatmap of the correlation between m^6^A regulators and tumor purity, as well as immune cell infiltration in the Cancer Genome Atlas (TCGA)-Colon adenocarcinoma (COAD) dataset. **(c, d)** Scatter plot showing a negative correlation between RBM15 expression and the Immune Score **(c)** as well as tumor purity **(d)**. **(e)** Box plot representing RBM15 expression levels in adjacent normal and tumor colon tissues. **(f)** Comparison of MSI MANTIS Scores between mutant RBM15 (Mutated) and Wild-Type (WT) colorectal cancer, showing a significant difference between the two groups. **(g)** Kaplan-Meier survival curves displays the survival probability over time (months) for two groups of patients: high RBM15 expression (red curve) and low RBM15 expression (black curve). The number of patients at risk at various time points is indicated below the plot. High RBM15 expression is associated with worse survival outcomes compared to low RBM15 expression. **(h)** Kaplan-Meier survival analysis of overall survival (OS) for advanced colorectal cancer patients treated with Bevacizumab, stratified by RBM15 expression into low (blue) and high (red) groups. The number of patients at risk at various time points is indicated below the plot.

To unravel the role of m^6^A modification in tumor immunomodulation of colorectal cancer, we analyzed immune scores based on the expression level of 141 genes reflecting immune signatures using the ESTIMATE platform (17). We selected a total of 19 m^6^A regulators, including writers, readers, and erasers, to access the correlation between their expression levels and immune scores (**Figure 1a**). In the Cancer Genome Atlas (TCGA)-Colon adenocarcinoma (COAD) dataset, *RBM15* RNA showed the second strongest inverse correlation with immune cell infiltration levels (**Figures 1b, c**). The immunomodulatory role of the top-ranked gene, YTHDC1, has been extensively investigated in other cancer types (18). Consistently, RBM15 expression was negatively correlated with tumor purity, suggesting RBM15 may suppress the recruitment of immune cells (**Figures 1b, d**).

We further explored whether RBM15 plays a role in immunomodulation in other digestive system cancers. Strikingly, *RBM15* RNA did not show a negative correlation with immune cell infiltration in liver hepatocellular carcinoma (LIHC), pancreatic adenocarcinoma (PAAD), or stomach adenocarcinoma (STAD) (**Supplementary Figures S1A-F**). Moreover, RBM15 expression was not correlated with tumor purity in LIHC or PAAD, but was negatively correlated with that in STAD (**Supplementary Figures S1G-I**). These findings indicate a colon tissue-specific oncogenic role of RBM15. Furthermore, RBM15 expression was significantly higher in colorectal cancer compared to adjacent normal tissues (**Figure 1E**).

Microsatellite instability (MSI) is a key predictor of responses to immunotherapy, in part because high MSI (MSI-H) tumors present foreign surface markers that are more easily recognized by immune cells (19). Indeed, colorectal cancer patients with MSI-H status tend to have better outcomes and often achieve a strong response to ICIs (5). We found that mutant RBM15 significantly linked to higher MSI MANTIS scores in colorectal cancer, supporting the role of RBM15 in regulating tumor immunomodulation (**Figure 1f**). Furthermore, high expression of RBM15 was associated with poor prognosis in colorectal cancer patients (**Figure 1g**). Notably, this association was more pronounced in advanced colorectal cancer patients receiving Bevacizumab treatment (**Figure 1h**). Overall, these findings suggest that RBM15 overexpression is negatively correlated with immune cell infiltration in colorectal cancer, presenting an immunosuppressive function of RBM15 in colorectal tumor microenvironment.

### RBM15 deficiency induces metabolic alterations in colorectal cancer

**Figure 2:**
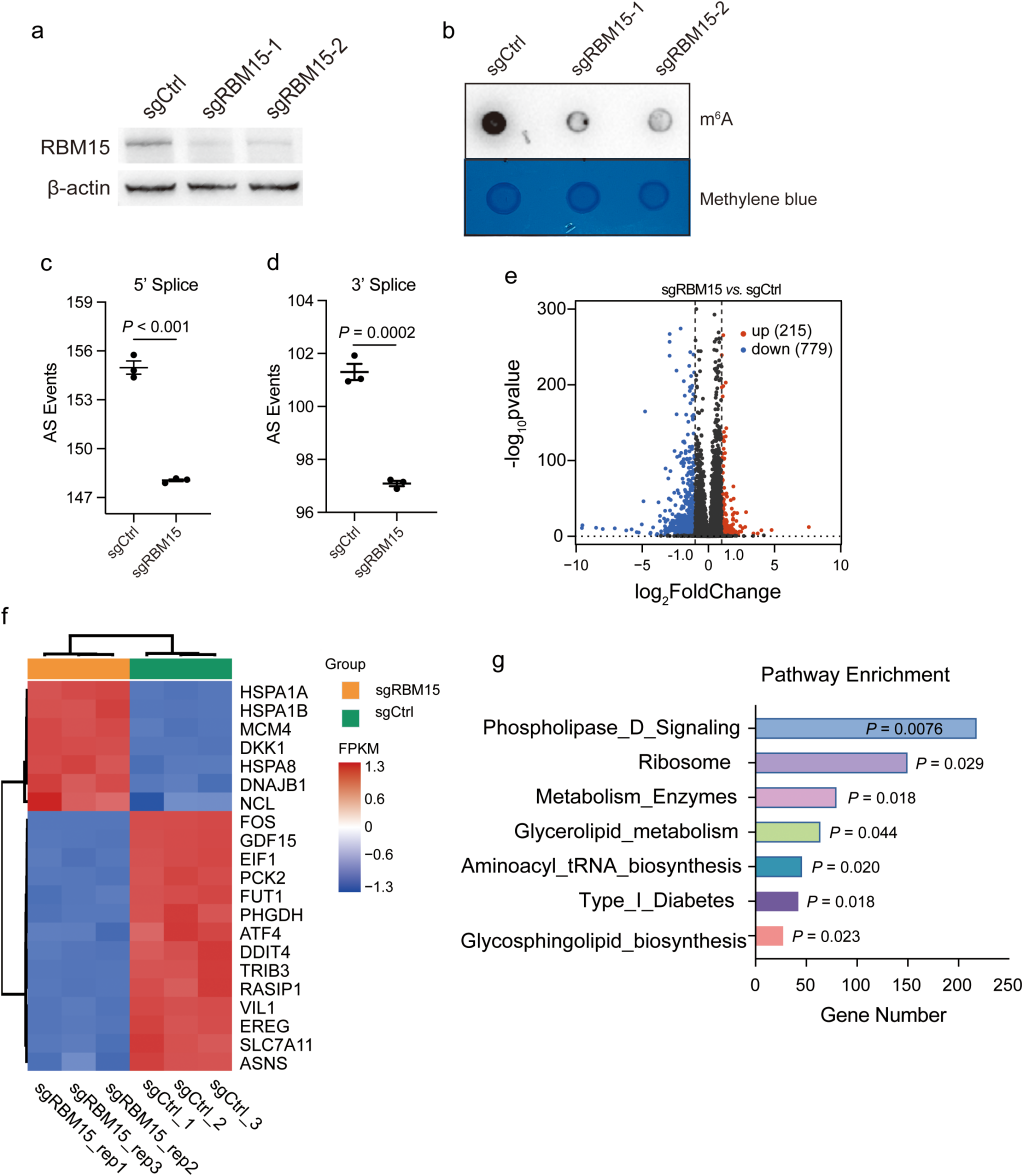
RBM15 deficiency induces metabolic alterations in colorectal cancer. **(a)** Western blot analysis showing RBM15 protein levels in the human colorectal cancer cell line HCT15 treated with control sgRNA (sgCtrl) and two different sgRNAs targeting RBM15 (sgRBM15-1 and sgRBM15-2). **(b)** Dot blot analysis showing m^6^A RNA methylation levels in HCT15 treated with control sgRNA (sgCtrl) and sgRNAs targeting RBM15 (sgRBM15-1 and sgRBM15-2). The methylene blue staining ensures equal loading across the samples. **(c, d)** Quantification of alternative splicing (AS) events at the 5' and 3' splice sites in control (sgCtrl) and RBM15 knockout (sgRBM15) of HCT15 cells. **(e)** Volcano plot illustrating differential gene expression between RBM15 KO (sgRBM15) and control (sgCtrl) HCT15 cells. Upregulated genes are marked in red (215 genes), while downregulated genes are marked in blue (779 genes). Non-significant genes are shown in black. **(f)** Heatmap representing top genes with differential expression between RBM15 KO (sgRBM15) and control (sgCtrl) HCT15 cells. The expression levels of selected genes (listed on the right) are shown as log-transformed Fragments Per Kilobase of transcript per Million mapped reads (FPKM) values. **(g)** Pathway enrichment analysis of differentially expressed genes, comparing the RBM15 KO (sgRBM15) and control (sgCtrl) group. .

Recent studies have highlighted the cancer cell-intrinsic mechanisms involved in modulating tumor immunity (20, 21). To reveal the tumor-intrinsic functional role of RBM15 in colorectal cancer, we initially utilized the CRISPR/Cas9 gene editing system to knock out (KO) RBM15 in the human colorectal cancer cell line HCT15. We designed two sgRNA fragments targeting the *RBM15* gene to minimize potential off-target effects. Western blotting analysis showed that RBM15 protein was significantly depleted (**Figure 2a**). Consistent with previous findings, we observed a marked reduction in m^6^A modification in the mRNA of RBM15 KO cells, further suggesting the sufficient KO efficiency and a shared functional consequence by the two distinct sgRNAs (**Figure 2b**).

To unravel the gene expression alterations induced by RBM15, we performed bulk RNA sequencing (RNA-seq) in RBM15 KO and wild-type (WT) colorectal cancer HCT15 cells. RBM15 KO significantly reduced alternative splicing events at both the 5’ and 3’ end of RNAs (**Figures 2c, d**). This was consistent with the previous finding that m^6^A modification regulates the alternative splicing of precursor RNAs (22, 23). Further analysis of differential gene expression revealed that a total of 994 genes were significantly altered (**Figure 2e**). Strikingly, genes associated with metabolic pathways were among the top differentially expressed genes, including the fucosyltransferase 1 (FUT1), the phosphoglycerate dehydrogenase (PHGDH), and the cystine transporter solute carrier family 7 member 11 (SLC7A11) (**Figure 2f**). Indeed, gene set enrichment analysis (GSEA) confirmed the enrichment of multiple pathways associated with metabolism (**Figure 2g**).

We further performed cell mitochondrial test to establish the mitochondrial function of the human and murine cells with and without RMB15. RBM15 knockout significantly decreased the maximal mitochondrial respiration in both human and murine colorectal cancer cells (**Supplementary Figure S2**). These findings suggest that RBM15 may regulate mitochondrial metabolism to affect cellular energy supply. In addition, RBM15 knockdown caused a slight reduction of cell proliferation in either human or murine colorectal cancer cells (**Supplementary Figure S3**).

### RBM15 depletion alters carbon metabolism and upregulates the expression of fumarate hydratase

To identify the fundamental metabolic pathways interfered by RBM15, we tracked the altered metabolites by RBM15 knockdown using untargeted high-resolution metabolic profiling. Orthogonal Partial Least Squares-Discriminant Analysis (OPLS-DA) revealed good reproducibility and discrimination for RBM15 knockdown and WT cells (**Supplementary Figures S4A, B**) (24). Through combining differential metabolites from both positive and negative ion modes, we identified a total of 860 significantly altered metabolites by RBM15 knockdown. We then categorized these altered metabolites based on their chemical taxonomy and showed that organic acids, lipids, and organ heterocyclic compounds accounted for the majority of the differentially expressed metabolites (**Supplementary Figures S4C**, **S5A-B**).

**Figure 3:**
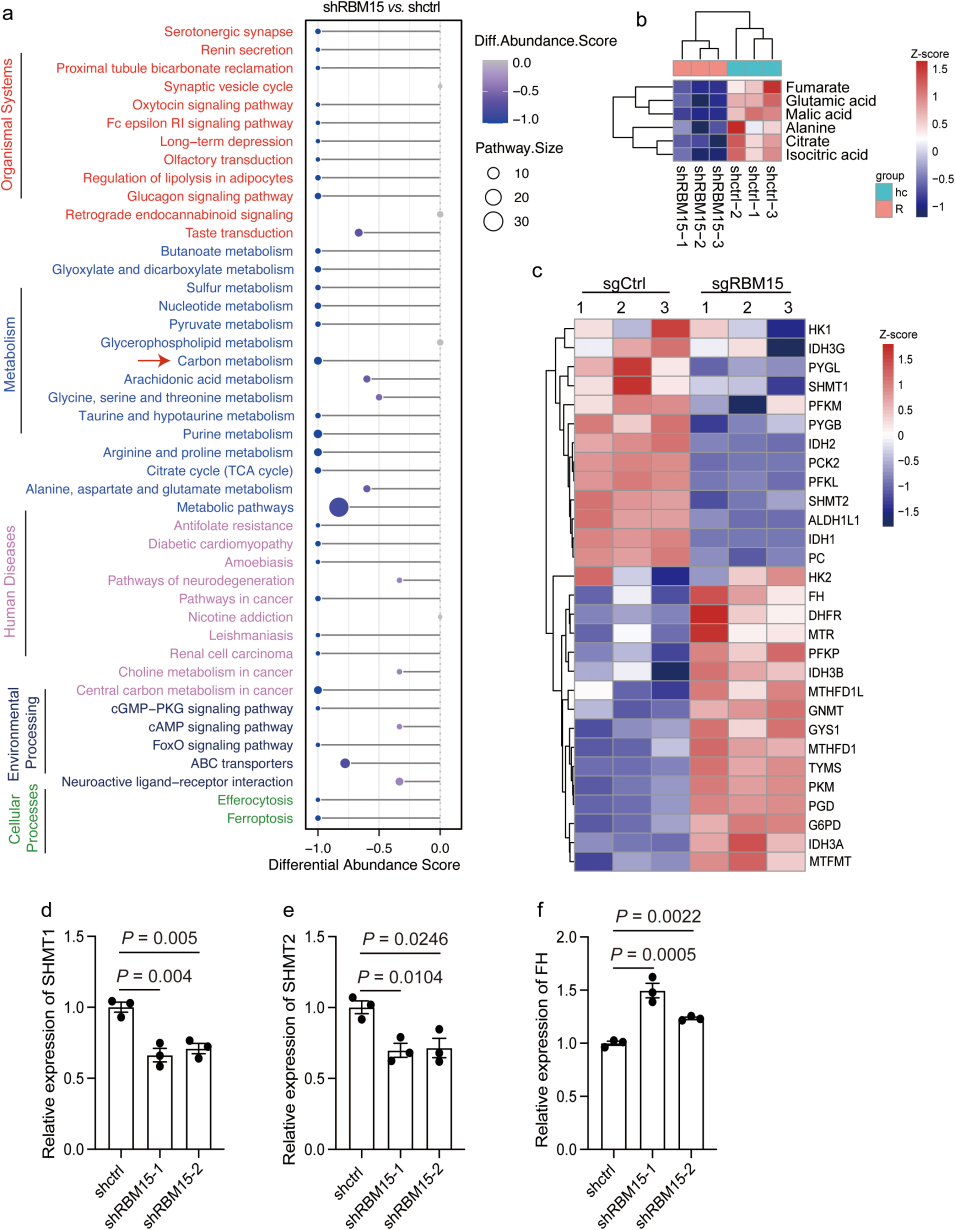
RBM15 depletion alters carbon metabolism and upregulates the expression of fumarate hydratase (FH). **(a)** Differential abundance score representing differentially downregulated pathways in cells with RBM15 knockdown (shRBM15) compared to control (shctrl) cells. The plot on the left displays the pathways grouped by functional categories. **(b)** Heatmap showing the *Z*-score normalized levels of specific metabolites associated with carbon metabolism including fumarate, glutamic acid, malic acid, alanine, citrate and isocitric acid, in cells with RBM15 knockdown (shRBM15) and control (shctrl) cells. **(c)** Heatmap depicting the *Z*-score normalized expression levels of metabolic enzymes involved in carbon metabolism in RBM15-knockout (sgRBM15) and control (sgCtrl) cells. **(d-f)** Bar graphs showing the relative expression levels of key catalytic enzymes involved in carbon metabolism, including SHMT1 **(d)**, SHMT2 **(e)**, and FH **(f)**, in RBM15-knockdown (shRBM15) and control (shctrl) cells. Data are represented as mean ± SEM. n = 3.

We next sought to determine the enriched differential metabolic pathway affected by RBM15 knockdown. Differential abundance analysis showed that carbon metabolism presented as one of the top downregulated metabolic pathways among all the enriched pathways matched from Kyoto Encyclopedia of Genes and Genomes (KEGG) (**Figure 3a**). We further examined the specific dysregulated metabolites associated with carbon metabolism by RBM15 knockdown. Metabolites including fumarate, glutamic acid, malic acid, alanine, citrate, and isocitric acid were significantly downregulated (**Figure 3b**). Accordingly, the expression levels of metabolic enzymes involved in carbon metabolism were altered due to RBM15 knockout, indicating a systematic reprogramming of cellular carbon metabolism caused by RBM15 depletion (**Figure 3c**). Furthermore, we confirmed by quantitative reverse transcriptase polymerase chain reaction (qRT-PCR) that the expression of key catalytic enzymes involved in carbon metabolism was significantly changed (**Figures 3d-f**).

Fumarate is produced through the carbon metabolism, and its accumulation in tumor interstitial fluid has been shown to suppress CD8+ T cell activation and anti-tumor immune responses (20). Conversely, fumarate depletion by increasing the expression of fumarate hydratase (FH) in tumor cells dramatically enhances the anti-tumor cytotoxicity of chimeric antigen receptor (CAR) T cells (20). Strikingly, we found that FH expression was significantly upregulated in human and murine colorectal cancer cells by RBM15 KO, which in turn led to a reduction in fumarate levels (**Figures 3b, c, f**, **Supplementary Figures S6A-C**). Collectively, these findings suggest that RBM15 depletion significantly affects carbon metabolism and upregulates the expression level of FH, which in turn downregulates fumarate in colorectal cancer.

### RBM15 deficiency delays tumor growth through enhanced immune infiltration

**Figure 4:**
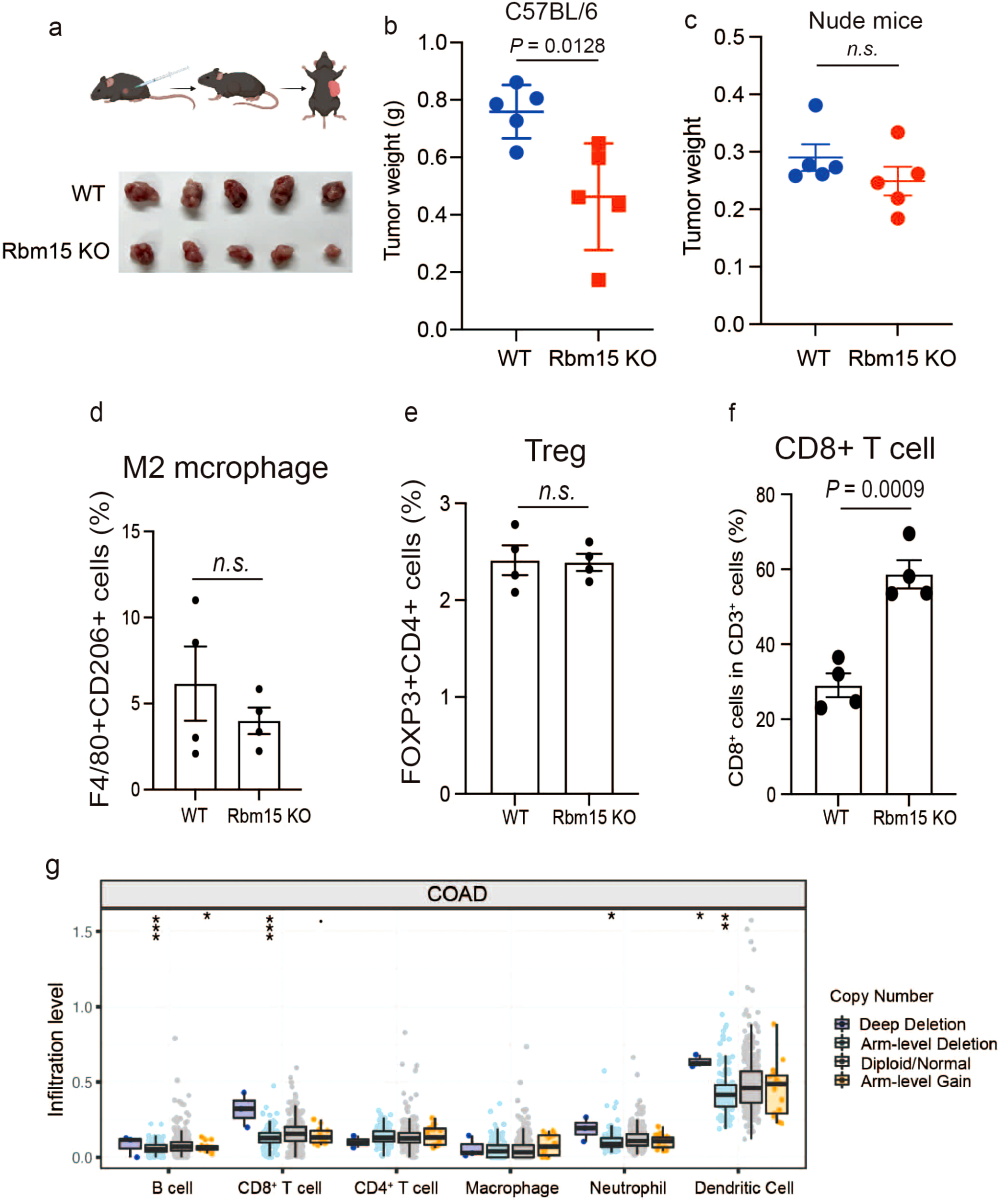
RBM15 deficiency delays tumor growth through enhanced immune infiltration. **(a)** Schematic illustration of subcutaneous injection of mouse syngeneic MC38 cells into immunocompetent C57BL/6J mice (upper panel) and tumor growth comparison between wild-type (WT) and Rbm15-knockout (Rbm15 KO) groups (lower panel). **(b)** Tumor weight in C57BL/6J mice comparing WT and Rbm15 KO groups. n=5 for each group. **(c)** Tumor weight in nude mice comparing WT and Rbm15 KO groups. n=5 for each group. **(d)** Representative flow cytometry analysis showing the expression of immune cell markers from the WT and Rbm15 KO tumors in C57BL/6J mice. **(d-f)** Quantitative flow cytometry analysis of immune cell populations within the immunocompetent tumor microenvironment of WT and Rbm15 KO mice. Analysis including the percentage of M2 macrophages (F4/80+CD206+ cells) **(d)**, Tregs (FOXP3+CD4+ cells) **(e)**, and CD8+ T cells **(f)**. **(g)** Infiltration levels of various immune cell types in colorectal adenocarcinoma (COAD) based on copy number alterations. Box plots show the infiltration levels of B cells, CD8+ T cells, CD4+ T cells, macrophages, neutrophils, and dendritic cells across groups with distinct copy number alterations, including deep deletion, arm-level deletion, diploid/normal, and arm-level gain. Statistical significance between groups is indicated (*P < 0.05, **P < 0.01, ***P < 0.001) n.s., non-significant.

Given that RBM15 depletion reduces fumarate levels, which could potentially enhance CD8+ T cell activation and increase anti-tumor immune responses, we next determine whether RBM15 deficiency could inhibit tumorigenesis via enhanced immune surveillance. We utilized the CRISPR/Cas9 gene editing system to deplete *Rbm15* gene in a synergetic mouse cell line MC38 (**Supplementary Figure S7A**). Then, immunocompetent C57BL/6J mice were subcutaneously injected with either *Rbm15*-KO cells or WT cells. Notably, Rbm15 knockout significantly prohibited tumorigenesis in immunocompetent mice, as evidenced by reductions in both tumor volume and weight, with no changes detected in body weights (**Figures 4a, b**, **Supplementary Figures S7B-C**). In contrast, there were limited differences in tumor weight between *Rbm15*-KO cells and WT cells in immunodeficient nude mice, suggesting that the reduced tumor growth caused by Rbm15 deficiency mainly attributed to the induction of anti-tumor immunity. (**Figures 4c**, **Supplementary Figure S8A**).

To investigate the alterations in the tumor immune microenvironment induced by RBM15, we performed multicolor flow cytometry analysis along with multiplex immunofluorescence (mIHC) to assess immune cell infiltration in mouse tumors (**Supplementary Figures S9**, **S10**). The results showed that Rbm15 knockout did not significantly alter the proportion of M2 macrophages (F4/80+/CD206+), Treg cells (FOXP3+/CD4+), or IFNγ production (**Figures 4d, e**, **Supplementary Figures S11A-B**, **S12A-C**). However, there were a significant increased proportion of CD8^+^ T cells, DCs (CD11C+), and TNF-α^+^ production (**Figures 4f**, **Supplementary Figures S11E-F**, **S12D**, **S13**). Consistently, the infiltration levels of CD8+ T cells and DCs were markedly higher in human colorectal cancer tumors with genetic alterations leading to deep depletion of RBM15 (**Figure 4g**). Furthermore, analysis of publicly available single-cell RNA datasets further demonstrated that there was a negative correlation between RBM15 expression and the CD8 T cell infiltration in tumors of colorectal cancer patients (**Supplementary Figure S14**). In addition, Rbm15 knockout significantly exhibited enhanced FH expression in MC38-derived xenograft tumors, demonstrating that RBM15 may regulate anti-tumor immune responses via FH modulation (**Supplementary Figure S15**). Overall, these findings suggest that RBM15 deficiency prohibits tumorigenesis, potentially through increased CD8+ T cell infiltration and activation.

## Discussion

Here we identify a cancer cell-intrinsic mechanism by which RBM15 suppresses the tumor immune response in colorectal cancer. Specifically, RBM15 deficiency significantly delayed colorectal tumor growth by enhancing immune cell infiltration, potentially due to reduced fumarate levels within the tumor microenvironment. This decrease in fumarate was linked to increased expression of fumarate hydratase (FH) induced by RBM15 depletion. Previous studies have highlighted the oncogenic role of RBM15 in various cancer types, such as breast and cervical cancer (12, 13). However, they did not address tumor immunity by focusing on immunocompromised environments. Our findings thus are significant because they connect the tumor-intrinsic functional role of RBM15 to the anti-tumor immune responses. Furthermore, we demonstrate that the increased infiltration of immune cells, such as CD8+ T cells, neutrophils, and dendritic cells, contributed to the enhanced anti-tumor immune responses. Nevertheless, whether CD8+ T cell infiltration is the dominant contributor to this response or whether it is part of a collective infiltration effect requires further investigation.

Tumor cells undergo metabolic rewiring to evade immune surveillance (25–27). Inhibitors targeting the cancer cell-intrinsic metabolic dysregulation have been shown to restore immunosurveillance, with several currently under development in clinical trials (21). However, increasing intrinsic fumarate levels through FH inhibition suppresses CD8+ T cell anti-tumor functions, making FH inhibitors unsuitable for exploring anti-tumor efficacy (20). Our study identifies RBM15 as an upstream regulator of FH, as RBM15 deficiency significantly increases FH expression while decreasing associated fumarate levels. Therefore, our findings suggest that RBM15 is a promising therapeutic target for enhancing anti-tumor immune responses through metabolic reprogramming. Currently, no specific inhibitors for RBM15 have been identified. However, with the recent elucidation of the crystal structure for human RBM15, it offers great potential to design, screen, or optimize inhibitors for future translational applications (10).

Accumulating evidences have bridged RNA modification, particularly m^6^A modification, to anti-tumor immunity (28–31). RBM15 is a key component of the m^6^A writer complex that specifically interacts with WTAP and VIRMA, both of which positively regulate m^6^A levels (10). Alteration in m^6^A levels of mRNAs broadly influence post-transcriptional regulation, such as mRNA stability and degradation (32). Beyond this, RBM15 contains phosphoserine binding modules that recognizes the C-terminal domain (CTD) of RNA polymerase II (Pol II), thereby synergizing with the m^6^A methyltransferase complex to mediate co-transcription (10). In our study, we revealed that RBM15 negatively regulated the expression of FH. However, further exploration is needed to determine whether this regulation occurs at the transcriptional level or through post-transcriptional mechanisms. Nevertheless, the connection between RBM15 and FH adds another layer to the understanding of anti-tumor immune responses, by which the m^6^A writer component suppresses the anti-tumor immunity through regulating key enzymes in carbon metabolism, leading to the release of immunosuppressive metabolites.

In summary, this study identifies a cancer cell-intrinsic mechanism by which RBM15 acts as a suppressor of anti-tumor immune responses through metabolic rewiring. This study also provides a compelling rationale for establishing RBM15 as a promising therapeutic target for colorectal cancer.

## Materials and methods

### Cell culture and reagents

The human colorectal cancer cell line HCT15 (Fuheng Bio, FH0026) and the mouse colorectal cancer cell line MC38 (LYNJUNE, LYN-0573) were cultured in RPMI-1640 medium (KGL1505-500) supplemented with 1% penicillin/streptomycin and 10% FBS (Gibco, A5669402) at 37°C in a 5% CO2 atmosphere. All cell lines were authenticated using short tandem repeat DNA profiling. Mycoplasma detection was performed monthly using a one-step mycoplasma detection kit (Vazyme, D201-01). CRISPR plasmids for inducing RBM15 knockout were purchased from Tsingke and utilized the pLentiCRISPR V2-puro backbone (Addgene #98290) with specific sgRNAs as insert fragments. The following sgRNAs were designed: human sgRBM15-1 (CCAGCTTAGTGACGAAGCGG), human sgRBM15-2 (GTGAAGGCCAAACGCTCCCG), and mouse sgRBM15-1 (GCGGCGCCGGCTCACGTACA). Additionally, lentiviral plasmids for RBM15 genetic knockdown were designed as follows with pLKO-puro as vector: human shRBM15-1 (GACGCCTTAGAGTAGACTTTG) and human shRBM15-2 (ATTACCTGGTCATGATCATTG).

### Lentivirus infection and selection

Lentiviral particles were produced for CRISPR knockout and genetic knockdown. Briefly, HEK293T cells (Fuheng, FH0244) were co-transfected with lentiviral plasmids and packaging plasmids pMD2.G and psPAX2 (Addgene #12260 and Addgene #12259) using the cationic polymer polyethylenimine (PEI, MW25000) at a ratio of 1:3. After 48 to 72 hours of transfection, the lentiviral particles were harvested from the culture supernatant by filtering through a 0.45 µm filter (Sangon, F513144). The harvested lentivirus was aliquoted and stored at −80°C until further use. HCT15 and MC38 colorectal cancer cells were infected with the lentivirus in the presence of 8 μg/ml polybrene (Biosharp, BL628A). Following transduction, 2 μg/ml puromycin (Meilun, MA0318) was used for 48 to 72 hours to select for knockout or knockdown cell lines.

### Western blot

Total protein was extracted from the indicated samples using RIPA buffer (50 mmol/L Tris-HCl (pH 8.0), 150 mmol/L NaCl, 1% Triton X-100, 0.5% sodium deoxycholate, 0.1% SDS, and 1 mmol/L phenylmethylsulfonyl fluoride) on ice for 20 minutes. The total protein concentration was measured using the BCA Protein Quantification Kit (Beyotime, P0012). Equal amounts of protein lysates were mixed with 1× loading buffer and heated at 95°C for 10 minutes. The samples were then loaded onto precast gels (GenScript, M00944) for electrophoresis. Following electrophoresis, proteins were transferred to a PVDF membrane (Millipore, ISEQ00010). The membrane was blocked with 5% bovine serum albumin (BSA) in Tris-buffered saline containing 0.1% Tween 20 (TBST) for 1 hour at room temperature, followed by overnight incubation at 4°C with the following primary antibodies: m^6^A mAb (Proteintech, 68055-1-Ig, 1:1000), RBM15 polyclonal antibody (Proteintech, 10587-1-Ab, 1:1000), and β-actin mAb (STARTER, S0B0005-100μg, 1:2000). After washing with TBST buffer, the membrane was incubated with horseradish peroxidase (HRP)–coupled secondary antibodies (CST, anti-rabbit #7074 and anti-mouse #7076, 1:3000) for 1 hour at room temperature. After washing three times with TBST buffer for 8 minutes each, protein bands were visualized using enhanced chemiluminescence (ECL) detection reagents (Millipore, WBKLS0500), and signals were detected using an Automatic ChemiDoc Imaging System (Tanon 5200).

### Quantitative real-time polymerase chain reaction

Total RNA was isolated from the samples using the FastPure Cell/Tissue Total RNA Isolation Kit (Vazyme, RC112-01) according to the manufacturer’s protocol. RNA concentration and purity were assessed using a NanoDrop One spectrophotometer (Thermo Fisher Scientific). Complementary DNA (cDNA) was synthesized from 1 μg of total RNA using HiScript II Q RT SuperMix for qPCR (Vazyme, R223-01). Quantitative PCR (qPCR) was performed using Taq Pro Universal SYBR qPCR Master Mix (Vazyme, Q712) on an QuantStudio Real-Time PCR machine with specific primers. The cycling conditions included an initial denaturation at 95°C for 5 minutes, followed by 40 cycles of denaturation at 95°C for 10 seconds and annealing at 60°C for 40 seconds. The specificity of the PCR products was confirmed by melting curve analysis. The relative transcriptional expression of target genes was normalized to the geometric mean of reference gene (B2M) and were evaluated by the comparative Ct (ΔΔCt) method. Fold changes were calculated using the 2^-ΔΔCt^ method. The following primers were used: human RBM15 For (ACGACCCGCAACAATGAAG), human RBM15 Rev (GGAAGTCGAGTCCTCACCAC), human B2M For (GAGGCTATCCAGCGTACTCCA), human B2M Rev (CGGCAGGCATACTCATCTTTT), human SHMT1 For (CTGGCACAACCCCTCAAAGA), human SHMT1 Rev (AGGCAATCAGCTCCAATCCAA), human SHMT2 For (CCCTTCTGCAACCTCACGAC), human SHMT2 Rev (TGAGCTTATAGGGCATAGACTCG), human FH For (GGAGGTGTGACAGAACGCAT), human FH Rev (CATCTGCTGCCTTCATTATTGC).

### Dot blot

Total RNA from the indicated samples was extracted using the FastPure Cell/Tissue Total RNA Isolation Kit (Vazyme, RC112-01). A final concentration of 1 µg/µl RNA was dotted onto a nitrocellulose (NC) membrane (SIMUWU, SD0045). The membrane was air-dried for 10 minutes and subsequently blocked with a 1% BSA (in PBST) solution for one hour. Methylene blue staining (0.2%, Yuanye Bio-Technology, R20768) was applied as a loading control. The membrane was then incubated with primary antibodies (m^6^A mAb, Proteintech, 68055-1-Ig, 1:1000) diluted in 1% BSA (in PBST) at room temperature for one hour. After washing four times with PBST buffer for 5 minutes each, the membrane was incubated with secondary antibodies (CST, #7076) diluted in 1% BSA in PBST at room temperature for one hour. Following four additional washes with PBST buffer for 5 minutes each, the membrane was treated with enhanced chemiluminescence (ECL) detection reagents (Millipore, WBKLS0500) and imaged using an Automatic ChemiDoc Imaging System (Tanon 5200).

### Cell proliferation assay

Cell proliferation was measured using the CellTiter-Lumi Cell Viability Assay (beyotime, #C0056). Cells were seeded in 96-well plates for 72 hours at indicated conditions. CellTiter-Lumi reagent was added to each well based on the manufacturers’ manual and luminescence was measured on a white microplate (beyotime, #FCP968) using a microplate reader (PerkinElmer). Luminescence values presented as mean ± SEM. Statistical significance was determined using Student’s t-test.

### RNA-sequencing and analysis

Total RNA from control HCT15 and RBM15-knockout HCT15 cell lines was extracted using TRIzol according to the manufacturer’s instructions (TSP401) across three independent groups. RNA sequencing (RNA-seq) libraries were constructed using the VAHTS Universal V6 RNA-seq Library Prep Kit for Illumina (NR604-02) and subjected to a paired-end 150 bp sequencing run on the Illumina NovaSeq 6000. Raw data were aligned using HISAT2 v2.2.1 against the hg38 version of the human genome, and read counts and fragments per kilobase million (FPKM) values for each sample were calculated using StringTie v2.0.4. The R package DESeq2 v1.26.0 was utilized to assess the significance of differential expression between group pairs and to calculate normalized counts. Gene expression changes were considered significant if they met the threshold of *P* < 0.05. Enrichment analysis was conducted using Gene Set Enrichment Analysis (GSEA v4.1.0), with results deemed significant at *P* < 0.05. Alternative splicing events in RNA-seq data were analyzed using ASprofile v1.0.4. Raw RNA-seq reads were aligned to the human genome (hg38) using HISAT2 (v2.2.1). ASprofile was used to detect and quantify alternative splicing events including 5’ splice site changes and 3’ splice site changes. Statistical analysis was performed using the Chi-square test (χ² test) with events considered significant if *P* < 0.05.

### Metabolic profiling

The cells were incubated and vortexed with a chilled extraction solution (2:2:1 v/v methanol/acetonitrile/water) for a minimum of 20 minutes. Subsequently, each sample was centrifuged at maximum speed at 4°C for 20 minutes, and the resulting supernatant was used for untargeted metabolomics analysis. The analysis was conducted using ultra-performance liquid chromatography coupled with a tandem quadrupole time-of-flight mass spectrometer (UHPLC-Q-TOF/MS). Chromatographic separation was achieved on an Agilent 1290 Infinity UHPLC system, with the column temperature maintained at 25°C, a flow rate of 0.3 mL/min, and an injection volume of 2 μL. The mobile phase consisted of solvent A: water + 25 mM ammonium acetate + 25 mM ammonia, and solvent B: acetonitrile. The gradient elution program was as follows: 0–1.5 min, 98% B; 1.5–12 min, B linearly decreased from 98% to 2%; 12–14 min, B was maintained at 2%; 14–14.1 min, B linearly increased from 2% to 98%; 14.1–17 min, B was maintained at 98%. Throughout the analysis, samples were kept in a 4°C autosampler. High-resolution tandem mass spectrometry was performed using Triple TOF 6600 spectrometers (AB SCIEX) under the following conditions: nebulizer gas (Gas1) at 60 psi, auxiliary gas (Gas2) at 60 psi, curtain gas (CUR) at 30 psi, ion source temperature at 600°C, and spray voltage (ISVF) at ±5500 V (for both positive and negative modes). The mass-to-charge ratio (m/z) range for the first stage was 80–1200 Da, with a resolution of 60,000 and a scan accumulation time of 100 ms. The second stage employed a segmented acquisition method, with a scan range of 70–1200 Da, a resolution of 30,000, a scan accumulation time of 50 ms, and a dynamic exclusion time of 4 seconds. Raw data were transformed into the “.mzXML” format using ProteoWizard. Finally, annotation and quantification of metabolites were performed using XCMS software version 3.7.1.

### Seahorse XF cell metabolism assay

To evaluate mitochondrial respiration, cells at indicated conditions were seeded in Seahorse XF 96-well plates (5×10⁴ cells per well) and incubated overnight. After the incubation in XF RPMI1640 base medium, the following metabolic modulators were sequentially injected: oligomycin, trifluoromethoxy carbonylcyanide phenylhydrazone (FCCP), and rotenone/antimycin A using XF Cell Mito Stress Test Kit (#103015-100). Oxygen consumption rate (OCR) was measured using the Seahorse XF Analyzer (Agilent Technologies) and was used to calculate maximal respiration. Each sample was normalized to protein quantity and presented as mean ± SEM.

### Flow cytometry analysis

For immune infiltration analysis, subcutaneous tumors were excised, minced into small pieces (1 to 2 mm), and digested using digestion buffer (abs9482). The cells were then filtered through 40 µm cell strainers. Analysis of tumor-infiltrating immune cells involved Live/Dead staining (BD, #564406), followed by Mouse Fc-blocking (BD, #553141), and surface staining in FACS buffer (BD, #554656) with fluorochrome-conjugated antibodies. The antibodies used included mouse CD45 (BD, #557659), mouse CD3 (BD, #553061), mouse CD8 (BD, #566985), mouse CD4 (BD, #550954), mouse CD11b (BD, #563015), and mouse F4/80 (BD, #565411), mouse FOXP3 (BD, #560408), mouse CD206 (BD, #568809), mouse TNFα (BD, #563943), and mouse IFNγ (BD, #561040). All FACS analyses were performed on a BD FACSCelesta, and the data were analyzed using FlowJo software.

### Animal experiments

Animal studies were approved by the Animal Care and Use Committee of Shanghai Jiao Tong University. Six- to eight-week-old mice were purchased from Hangzhou Ziyuan Experimental Animal Technology Co., Ltd. A total of 1 × 10^6 MC38 cells, with or without Rbm15 knockout, were subcutaneously injected into the right flank of male C57BL/6 or nude mice (n = 5 mice per group). Tumor volume, tumor weight, and body weight of the mice were measured at specified time points.

### Data mining

The immune score was calculated using the ESTIMATE platform, an approach that provides with scores for tumor purity, and the infiltration level of immune cells in tumor tissues based on expression data from The Cancer Genome Atlas (TCGA) tumors (17). Pearson correlation was used for calculating *P* and R values. Differential gene expression analysis in colorectal tumor vs normal tissues were performed by the TNMplot platform (33). MSI MANTIS score was calculated using the cBioPortal platform (34). The correlation between the expression of RBM15 and survival in colorectal patients were performed using Kaplan-Meier Plotter with probe datasets as1555760_a_at and cBioPortal (Colorectal Adenocarcinoma, TCGA, PanCancer Atlas). The abundances of six immune infiltrates (B cells, CD4+ T cells, CD8+ T cells, Neutrophils, Macrophages, and Dendritic cells) are calculated from the TIMER platform (35).

### Statistics and reproducibility

For statistical analysis, experiments were conducted at least three times, unless otherwise specified. Statistical analyses were performed using GraphPad Prism 7 software. The significance of differences between groups was assessed using a two-tailed unpaired Student’s t-test. Quantitative data are presented as mean ± SEM. A significance threshold of *P* < 0.05 was used for all statistical analyses.

## Data Availability

The bulk-RNA sequencing data will is available on the Gene Expression Omnibus (GSE279678).

## References

[1] Roshandel G Ghasemi-Kebria F Malekzadeh R. Colorectal cancer: epidemiology, risk factors, and prevention. Cancers (Basel). (2024) 16. doi: 10.3390/cancers16081530 PMC11049480 38672612

[2] Siegel RL Wagle NS Cercek A Smith RA Jemal A. Colorectal cancer statistics, 2023. CA Cancer J Clin. (2023) 73:233–54. doi: 10.3322/caac.21772 36856579

[3] Golshani G Zhang Y. Advances in immunotherapy for colorectal cancer: a review. Therap Adv Gastroenterol. (2020) 13:1756284820917527. doi: 10.1177/1756284820917527 PMC7268115 32536977

[4] Ros J Baraibar I Saoudi N Rodriguez M Salva F Tabernero J. Immunotherapy for colorectal cancer with high microsatellite instability: the ongoing search for biomarkers. Cancers (Basel). (2023) 15. doi: 10.3390/cancers15174245 PMC10486610 37686520

[5] Ganesh K Stadler ZK Cercek A Mendelsohn RB Shia J Segal NH. Immunotherapy in colorectal cancer: rationale, challenges and potential. Nat Rev Gastroenterol Hepatol. (2019) 16:361–75. doi: 10.1038/s41575-019-0126-x PMC7295073 30886395

[6] Winkler R Gillis E Lasman L Safra M Geula S Soyris C. m(6)A modification controls the innate immune response to infection by targeting type I interferons. Nat Immunol. (2019) 20:173–82. doi: 10.1038/s41590-018-0275-z 30559377

[7] Han D Liu J Chen C Dong L Liu Y Chang R. Anti-tumour immunity controlled through mRNA m(6)A methylation and YTHDF1 in dendritic cells. Nature. (2019) 566:270–4. doi: 10.1038/s41586-019-0916-x PMC6522227 30728504

[8] Lin W Chen L Zhang H Qiu X Huang Q Wan F. Tumor-intrinsic YTHDF1 drives immune evasion and resistance to immune checkpoint inhibitors via promoting MHC-I degradation. Nat Commun. (2023) 14:265. doi: 10.1038/s41467-022-35710-7 36650153 PMC9845301

[9] Cao Y Qiu G Dong Y Zhao W Wang Y. Exploring the role of m (6) A writer RBM15 in cancer: a systematic review. Front Oncol. (2024) 14:1375942. doi: 10.3389/fonc.2024.1375942 38915367 PMC11194397

[10] Appel LM Franke V Benedum J Grishkovskaya I Strobl X Polyansky A. The SPOC domain is a phosphoserine binding module that bridges transcription machinery with co- and post-transcriptional regulators. Nat Commun. (2023) 14:166. doi: 10.1038/s41467-023-35853-1 36631525 PMC9834408

[11] Liang Y Zhong H Zhao Y Tang X Pan C Sun J. Epigenetic mechanism of RBM15 in affecting cisplatin resistance in laryngeal carcinoma cells by regulating ferroptosis. Biol Direct. (2024) 19:57. doi: 10.1186/s13062-024-00499-6 39039611 PMC11264397

[12] Park SH Ju JS Woo H Yun HJ Lee SB Kim SH. The m(6)A writer RBM15 drives the growth of triple-negative breast cancer cells through the stimulation of serine and glycine metabolism. Exp Mol Med. (2024) 56:1373–87. doi: 10.1038/s12276-024-01235-w PMC11263342 38825643

[13] Wang R Tan W. RBM15-mediated N6-methyl adenosine (m^6^A) modification of EZH2 drives the epithelial-mesenchymal transition of cervical cancer. Crit Rev Eukaryot Gene Expr. (2024) 34:15–29. doi: 10.1615/CritRevEukaryotGeneExpr.2024052205 38842201

[14] Xia L Oyang L Lin J Tan S Han Y Wu N. The cancer metabolic reprogramming and immune response. Mol Cancer. (2021) 20:28. doi: 10.1186/s12943-021-01316-8 33546704 PMC7863491

[15] Chang CH Qiu J D Buck MD Noguchi T Curtis JD. Metabolic competition in the tumor microenvironment is a driver of cancer progression. Cell. (2015) 162:1229–41. doi: 10.1016/j.cell.2015.08.016 PMC4864363 26321679

[16] Thompson EA Powell JD. Inhibition of the adenosine pathway to potentiate cancer immunotherapy: potential for combinatorial approaches. Annu Rev Med. (2021) 72:331–48. doi: 10.1146/annurev-med-060619-023155 PMC8074264 32903139

[17] Yoshihara K Shahmoradgoli M Martinez E Vegesna R Kim H Torres-Garcia W. Inferring tumour purity and stromal and immune cell admixture from expression data. Nat Commun. (2013) 4:2612. doi: 10.1038/ncomms3612 24113773 PMC3826632

[18] Yan H Zhang L Cui X Zheng S Li R. Roles and mechanisms of the m(6)A reader YTHDC1 in biological processes and diseases. Cell Death Discovery. (2022) 8:237. doi: 10.1038/s41420-022-01040-2 35501308 PMC9061745

[19] Boland CR Goel A. Microsatellite instability in colorectal cancer. Gastroenterology. (2010) 138:2073–2087 e3. doi: 10.1053/j.gastro.2009.12.064 20420947 PMC3037515

[20] Cheng J Yan J Liu Y Shi J Wang H Zhou H. Cancer-cell-derived fumarate suppresses the anti-tumor capacity of CD8(+) T cells in the tumor microenvironment. Cell Metab. (2023) 35:961–978 e10. doi: 10.1016/j.cmet.2023.04.017 37178684

[21] De Martino M Rathmell JC Galluzzi L Vanpouille-Box C. Cancer cell metabolism and antitumour immunity. Nat Rev Immunol. (2024) 24:654–69. doi: 10.1038/s41577-024-01026-4 PMC11365797 38649722

[22] Rong S Dai B Yang C Lan Z Wang L Xu L. HNRNPC modulates PKM alternative sp*licing via* m^6^A methylation, upregulating PKM2 expression to promote aerobic glycolysis in papillary thyroid carcinoma and drive Malignant progression. J Transl Med. (2024) 22:914. doi: 10.1186/s12967-024-05668-9 39380010 PMC11459990

[23] Zhu ZM Huo FC Zhang J Shan HJ Pei DS. Crosstalk between m^6^A modification and alternative sp*licing during cancer progression* . Clin Transl Med. (2023) 13:e1460. doi: 10.1002/ctm2.v13.10 37850412 PMC10583157

[24] Worley B Powers R. PCA as a practical indicator of OPLS-DA model reliability. Curr Metabolomics. (2016) 4:97–103. doi: 10.2174/2213235X04666160613122429 27547730 PMC4990351

[25] Nicolini A Ferrari P. Involvement of tumor immune microenvironment metabolic reprogramming in colorectal cancer progression, immune escape, and response to immunotherapy. Front Immunol. (2024) 15:1353787. doi: 10.3389/fimmu.2024.1353787 39119332 PMC11306065

[26] Agarwala Y Brauns TA Sluder AE Poznansky MC Gemechu Y. Targeting metabolic pathways to counter cancer immunotherapy resistance. Trends Immunol. (2024) 45:486–94. doi: 10.1016/j.it.2024.05.006 38876831

[27] Zhou W Liu H Yuan Z Zundell J Towers M Lin J. Targeting the mevalonate pathway suppresses ARID1A-inactivated cancers by promoting pyroptosis. Cancer Cell. (2023) 41:740–756 e10. doi: 10.1016/j.ccell.2023.03.002 36963401 PMC10085864

[28] Shi JX Zhang ZC Yin HZ Piao XJ Liu CH Liu QJ. RNA m^6^A modification in ferroptosis: implications for advancing tumor immunotherapy. Mol Cancer. (2024) 23:213. doi: 10.1186/s12943-024-02132-6 39342168 PMC11437708

[29] Guo L Yang H Zhou C Shi Y Huang L Zhang J. N6-methyladenosine RNA modification in the tumor immune microenvironment: novel implications for immunotherapy. Front Immunol. (2021) 12:773570. doi: 10.3389/fimmu.2021.773570 34956201 PMC8696183

[30] Li X Ma S Deng Y Yi P Yu J. Targeting the RNA m(6)A modification for cancer immunotherapy. Mol Cancer. (2022) 21:76. doi: 10.1186/s12943-022-01558-0 35296338 PMC8924732

[31] Qu Y Gao N Zhang S Gao L He B Wang C. Role of N6-methyladenosine RNA modification in cancer. MedComm. (2020) 5:e715. doi: 10.1002/mco2.715 PMC11381670 39252821

[32] Jiang X Liu B Nie Z Duan L Xiong Q Jin Z. The role of m^6^A modification in the biological functions and diseases. Signal Transduction Targeted Ther. (2021) 6. doi: 10.1038/s41392-020-00450-x PMC7897327 33611339

[33] Bartha A Gyorffy B. TNMplot.com: A web tool for the comparison of gene expression in normal, tumor and metastatic tissues. Int J Mol Sci. (2021) 22. doi: 10.3390/ijms22052622 PMC7961455 33807717

[34] Cerami E Gao J Dogrusoz U Gross BE Sumer SO Aksoy BA. The cBio cancer genomics portal: an open platform for exploring multidimensional cancer genomics data. Cancer Discovery. (2012) 2:401–4. doi: 10.1158/2159-8290.CD-12-0095 PMC3956037 22588877

[35] Li T Fan J Wang B Traugh N Chen Q Liu JS. TIMER: A web server for comprehensive analysis of tumor-infiltrating immune cells. Cancer Res. (2017) 77:e108–10. doi: 10.1158/1538-7445.AM2017-108 PMC6042652 29092952

