# Supplementary material for: RBM15-mediated metabolic reprogramming boosts immune response in colorectal cancer
Source: Front Immunol. 2025 Apr 30;16:1515568. doi: 10.3389/fimmu.2025.1515568 (PMC12075365; doi:10.3389/fimmu.2025.1515568)
Supplement: Supplementary file 1 [file DataSheet1.pdf]

Figure 1. RBM15 overexpression is negatively correlated with immune cell infiltration in colorectal cancer. (a) A conceptual diagram illustrating the strategy to identify potential correlation between the expression levels of m^6A regulators and tumor purity, as well as immune cell infiltration. (b) Heatmap of the correlation between m^6A regulators and tumor purity, as well as immune cell infiltration in the Cancer Genome Atlas (TCGA)-Colon adenocarcinoma (COAD) dataset. (c-d) Scatter plot showing a negative correlation between RBM15 expression and the Immune Score (c) as well as tumor purity (d). (e) Box plot representing RBM15 expression levels in adjacent normal and tumor colon tissues. (f) Comparison of MSI MANTIS Scores between mutant RBM15 (Mutated) and Wild-Type (WT) colorectal cancer, showing a significant difference between the two groups. (g) Kaplan-Meier survival curves displays the survival probability over time (months) for two groups of patients: high RBM15 expression (red curve) and low RBM15 expression (black curve). The number of patients at risk at various time points is indicated below the plot. High RBM15 expression is associated with worse survival outcomes compared to low RBM15 expression. (h) Kaplan-Meier survival analysis of overall survival (OS) for advanced colorectal cancer patients treated with Bevacizumab, stratified by RBM15 expression into low (blue) and high (red) groups. The number of patients at risk at various time points is indicated below the plot.

Figure 2. RBM15 deficiency induces metabolic alterations in colorectal cancer. (a) Western blot analysis showing RBM15 protein levels in the human colorectal cancer cell line HCT15 treated with control sgRNA (sgCtrl) and two different sgRNAs targeting RBM15 (sgRBM15-1 and sgRBM15-2). (b) Dot blot analysis showing m^6A RNA methylation levels in HCT15 treated with control sgRNA (sgCtrl) and sgRNAs targeting RBM15 (sgRBM15-1 and sgRBM15-2). The methylene blue staining ensures equal loading across the samples. (c-d) Quantification of alternative splicing (AS) events at the 5' and 3' splice sites in control (sgCtrl) and RBM15 knockout (sgRBM15) of HCT15 cells. (e) Volcano plot illustrating differential gene expression between RBM15 KO (sgRBM15) and control (sgCtrl) HCT15 cells. Upregulated genes are marked in red (215 genes), while downregulated genes are marked in blue (779 genes). Non-significant genes are shown in black. (f) Heatmap representing top genes with differential expression between RBM15 KO (sgRBM15) and control (sgCtrl) HCT15 cells. The expression levels of selected genes (listed on the right) are shown as log-transformed Fragments Per Kilobase of transcript per Million mapped reads (FPKM) values. (g) Pathway enrichment analysis of differentially expressed genes, comparing the RBM15 KO (sgRBM15) and control (sgCtrl) group.

Figure 3. RBM15 depletion alters carbon metabolism and upregulates the expression of fumarate hydratase (FH). (a) Differential abundance score representing differentially downregulated pathways in cells with RBM15

knockdown (shRBM15) compared to control (shctrl) cells. The plot on the left displays the pathways grouped by functional categories. (b) Heatmap showing the *Z*-score normalized levels of specific metabolites associated with carbon metabolism including fumarate, glutamic acid, malic acid, alanine, citrate and isocitric acid, in cells with RBM15 knockdown (shRBM15) and control (shctrl) cells. (c) Heatmap depicting the *Z*-score normalized expression levels of metabolic enzymes involved in carbon metabolism in RBM15-knockout (sgRBM15) and control (sgCtrl) cells. (d-f) Bar graphs showing the relative expression levels of key catalytic enzymes involved in carbon metabolism, including SHMT1 (d), SHMT2 (e), and FH (f), in RBM15-knockdown (shRBM15) and control (shctrl) cells. Data are represented as mean ± SEM. n = 3.

Figure 4. RBM15 deficiency delays tumor growth through enhanced immune infiltration. (a) Schematic illustration of subcutaneous injection of mouse syngeneic MC38 cells into immunocompetent C57BL/6J mice (upper panel) and tumor growth comparison between wild-type (WT) and Rbm15-knockout (Rbm15 KO) groups (lower panel). (b) Tumor weight in C57BL/6J mice comparing WT and Rbm15 KO groups. n=5 for each group. (c) Tumor weight in nude mice comparing WT and Rbm15 KO groups. n=5 for each group. (d) Representative flow cytometry analysis showing the expression of immune cell markers from the WT and Rbm15 KO tumors in C57BL/6J mice. (d-f) Quantitative flow cytometry analysis of immune cell populations within the immunocompetent tumor microenvironment of WT and Rbm15 KO mice. Analysis including the percentage of M2 macrophages (F4/80+CD206+ cells) (d), Tregs (FOXP3+CD4+ cells) (e), and CD8+ T cells (f). (g) Infiltration levels of various immune cell types in colorectal adenocarcinoma (COAD) based on copy number alterations. Box plots show the infiltration levels of B cells, CD8+ T cells, CD4+ T cells, macrophages, neutrophils, and dendritic cells across groups with distinct copy number alterations, including deep deletion, arm-level deletion, diploid/normal, and arm-level gain. Statistical significance between groups is indicated (*$^*P < 0.05$, $^{**}P < 0.01$, $^{***}P < 0.001$*).

Figure S1. A colorectal cancer-specific role of RBM15. (a-c) The heatmaps depict the correlation between the expression levels of m^6A regulators and immune cell infiltration or tumor purity in liver hepatocellular carcinoma (LIHC) (a), pancreatic adenocarcinoma (PAAD) (b), and stomach adenocarcinoma (STAD) (c). (d-f) Scatter plot showing correlation between RBM15 expression and immune cell infiltration (Immune Score) in LIHC (d), PAAD (e) and STAD (f). (g-i) Scatter plot showing correlation between RBM15 expression and tumor purity (purity Score) in LIHC (g), PAAD (h) and STAD (i).

Figure S2. RBM15 knockout reduces mitochondrial respiration in colorectal cancer cells. (a-d) The Seahorse XF cell energy metabolism analysis was used to assess the oxygen consumption rate (OCR) in human

HCT15 (a) and murine MC38 (c) cells from the control group (sgCtrl) and RBM15/Rbm15 knockout group. Mitochondrial respiration parameters were determined by sequential addition of Oligomycin (ATP synthase inhibitor), FCCP (mitochondrial uncoupler), and Rotenone & Antimycin A (inhibitors of electron transport chain complexes I and III). Quantification of the maximal respiration capacity (Maximal Respiration) in HCT15 (b) and MC38 (d) cells from the control group (sgCtrl) and RBM15 knockout group (sgRBM15/sgRbm15). Data are presented as mean ± SEM. n=3.

Figure S3. Effect of RBM15 depletion on cell proliferation activity in colorectal cancer cells. (a) Luminescence measurements of HCT15 cells with control (shctrl) or RBM15 knockdown (shRBM15-1, shRBM15-2). (b) Luminescence measurements of HCT116 cells with control (shctrl) or RBM15 knockdown (shRBM15-1, shRBM15-2). (c) Luminescence measurements of MC38 cells with Rbm15 knockout (sgRbm15) or control (sgctrl). (d) Luminescence measurements of CT26 cells with Rbm15 knockout (sgRbm15) or control (sgctrl). Data are presented as mean ± SEM. n=3.

Figure S4. RBM15 deficiency led to metabolic alteration. (a) Quantitative analysis of RBM15 expression levels in control (shctrl) cells or RBM15-knockdown HCT15 cells (shRBM15-1 and shRBM15-2). Data represented as mean ± SEM. n = 3 for each group. (b) Orthogonal Partial Least Squares Discriminant Analysis (OPLS-DA) score for control and RBM15-knockdown HCT15 cells. The OPLS-DA plot shows separation between shctrl (purple) and shRBM15 (green) groups based on untargeted high-resolution metabolic profiling. (c) Distribution of metabolite classes in 860 significantly altered metabolites by RBM15 knockdown. The pie chart represents the percentage of metabolites classified into different classes.

Figure S5. Differential metabolite expression by RBM15 depletion. (a-b) Differential metabolite expression in positive (a) or negative (b) ion mode by RBM15 knockdown. The bar chart illustrates the log2 fold change (log2FC) of metabolites between control and RBM15-knockdown groups, with specific metabolites listed on the y-axis. Green bars indicate downregulated metabolites, while red bars indicate upregulated metabolites. The metabolites are grouped into different classes, represented by color-coded labels.

Figure S6. Effect of RBM15 depletion on fumarate hydratase (FH) expression in colorectal cancer cell lines. (a) Relative expression of *FH* in HCT116 cells with control (shctrl) or RBM15 knockdown (shRBM15-1, shRBM15-2). (b) Relative expression of *Fh* in MC38 cells with Rbm15 knockout (sgRbm15) or control (sgctrl). (c) Relative expression of *Fh* in CT26 cells with Rbm15 knockout (sgRbm15) or control (sgctrl). Data are presented as mean ± SEM. n=3.

Figure S7. RBM15 deficiency delays tumor growth. (a) Western blot analysis of Rbm15 expression in WT and Rbm15-knockout (Rbm15 KO) synergetic mouse MC38 cells. (b) Tumor growth curves comparing WT and Rbm15 KO in immunocompetent C57BL/6J mice for 16 days. (c) Body weight measurements between WT and Rbm15 KO groups in immunocompetent C57BL/6J mice for 16 days.

Figure S8. Tumor growth of MC38 cells in nude mice with Rbm15 knockout. (a) Tumor growth curves comparing WT (sgCtrl) and Rbm15 KO (sgRbm15) in immunodeficient nude mice. Data are presented as mean ± SEM. n=5.

Figure S9. Gating strategy 1 for flow cytometry (FACS) analysis of tumor-infiltrating immune cells. (a) Flow cytometry was used to analyze immune cell populations in mouse tumor tissues. Forward scatter (FSC-A) and side scatter (SSC-A) were used to select the cell population, and doublets were excluded by using FSC-H vs. FSC-A, obtaining single-cell populations. Live/Dead staining was used to exclude dead cells, selecting the live cell population. $CD45^+$ was used to identify tumor-infiltrating leukocytes (TILs). Among the $CD45^+$ cell population, further differentiation was made between $CD3^+$ T cells and $CD3^-$ myeloid cells. Among the $CD3^+$ cell population, $CD4^+$ T cells and $CD8^+$ cytotoxic T cells were distinguished. In the $CD3^-$ cell population, F4/80 and CD11b were used to distinguish macrophages from other myeloid cells.

Figure S10. Gating strategy 2 for flow cytometry (FACS) analysis of tumor-infiltrating immune cell subsets. (a) Flow cytometry was used to sort and analyze immune cell subsets in mouse tumor tissues. First, forward scatter (FSC-A) and side scatter (SSC-A) were used to select the cell population, and doublets were excluded using FSC-H vs. FSC-A, obtaining single-cell populations. Live/Dead staining was used to exclude dead cells and select live cell populations. $CD45^+$ was used to select tumor-infiltrating leukocytes (TILs). Within the $CD45^+$ population, further differentiation was made between $CD3^+$ T cells and $CD3^-CD11b^+$ myeloid cells. Among the $CD3^+$ T cell population, $CD4^+Foxp3^+$ regulatory T cells (Tregs) were identified. Among the $CD3^-CD11b^+$ myeloid cell population, $F4/80^+CD206^+$ M2 macrophages were distinguished. Additionally, the $CD45^+$ cell population was analyzed for IFN-γ production and TNF-α production.

Figure S11. Effect of RBM15 knockout on immune cell subsets in tumor tissues. (q, b) Flow cytometry analysis of the percentage of $F4/80^+CD11b^+$ cells (macrophages) in CD3- cells from WT group (a) and Rbm15 KO group (b). (c, d) Flow cytometry analysis of the percentage of $CD4^+$ cells in CD3+ cells from WT group (c) and Rbm15 KO group (d). (e, f) Flow cytometry analysis of the percentage of $CD8^+$ cells in CD3+ cells from WT group (e) and Rbm15 KO

group (f).

Figure S12. Effect of RBM15 knockout on immune cell infiltration in the tumor microenvironment. (a) Flow cytometry analysis of the percentage of M2 macrophages (F4/80$^+$CD206$^+$ cells) in CD3-CD11b+ cells from WT group and Rbm15 KO group. (b) Flow cytometry analysis of the percentage of regulatory T cells (Tregs, FOXP3$^+$ CD4$^+$ cells) in CD3+CD11b- from WT group and Rbm15 KO group. (c) Flow cytometry analysis IFN-γ$^+$ production in CD45+ cells from WT group and Rbm15 KO group. The bar chart shows IFN-γ$^+$ production in each group. (d) Flow cytometry analysis TNF-α$^+$ production in CD45+ cells from WT group and Rbm15 KO group. The bar chart shows TNF-α$^+$ production in each group. The bar chart data are presented as mean ± SEM. n=4.

Figure S13. RBM15 knockout affects CD8$^+$ and CD11c$^+$ cell infiltration in tumor tissues. (a) Immunofluorescence (IF) staining analysis of tumor tissues from WT (sgctrl) and Rbm15 KO (sgRbm15) group, showing CD8$^+$ cells (yellow) and CD11c$^+$ cells (DCs, red). DAPI was used to stain cell nuclei (blue). (b) Box plots showing the percentage of CD8$^+$ cells (left) and CD11c$^+$ cells (right) in the WT (sgctrl) and Rbm15 KO (sgRbm15) groups. Data are presented as mean ± SEM. n=4.

Figure S14. Analysis of RBM15 expression and immune cell infiltration in colorectal cancer using single-cell RNA sequencing (scRNA-seq) data. (a) Representative figure from pubilicly available scRNA-seq datasets (GSE245552) with low RBM15 expression in epithelial cells (Patient 17). UMAP plot showing the distribution of various cell types in the colorectal cancer samples, with cell types labeled accordingly. (b) UMAP plot of RBM15 expression levels across the cell types, with color intensity indicating the expression level (Patient 17). (c) Representative figure with high RBM15 expression in epithelial cells (Patient 8). UMAP plot showing the distribution of cell types in the dataset, including various immune cells, epithelial cells, and other cell types. (d) UMAP plot of RBM15 expression across all cell types, showing the distribution of RBM15 expression (Patient 8). (e-f) Pearson correlation analysis of RBM15 with CD8A (e, r = -0.22) and CD8B (f, r = -0.199) in the GSE245552 dataset (n = 39).

Figure S15. Effect of RBM15 knockout on FH expression in colorectal tumor tissues. (a) Immunohistochemistry (IHC) analysis of FH expression in tumor tissues from WT and Rbm15 KO groups. Brown staining represents FH-positive cells. (b) Quantification of the percentage of FH$^+$ cells in WT and Rbm15 KO groups. Data are presented as mean ± SEM. n=11 fields from three staining mouse tumor tissues.
